# Supplementary material for: Strong chemotaxis by marine bacteria towards polysaccharides is enhanced by the abundant organosulfur compound DMSP
Source: Nat Commun. 2023 Dec 6;14:8080. doi: 10.1038/s41467-023-43143-z (PMC10700628; doi:10.1038/s41467-023-43143-z)
Supplement: Supplementary file 1 — Supplementary Information [file 41467_2023_43143_MOESM1_ESM.docx]

**Supplementary Information**

**Strong chemotaxis by marine bacteria towards polysaccharides**

**is enhanced by the abundant organosulfur compound DMSP**

Estelle E. Clerc^1^, Jean-Baptiste Raina^2^*, Johannes M. Keegstra^1^, Zachary Landry^1^, Sammy Pontrelli^3^, Uria Alcolombri^1,4^, Bennett S. Lambert^1^, Valerio Anelli^1^, Flora Vincent^5^, Marta Masdeu-Navarro^6^, Andreas Sichert^7^, Frédéric De Schaetzen^1^, Rafel Simó^6^, Jan-Hendrik Hehemann^7^, Assaf Vardi^5^, Justin R. Seymour^2^ & Roman Stocker^1^*

^1^ Institute of Environmental Engineering, Department of Civil, Environmental and Geomatic Engineering, ETH Zurich, Zurich, Switzerland.

^2^ Climate Change Cluster, University of Technology Sydney, Ultimo, Australia.

^3^ Institute of Molecular Systems Biology, Department of Biology, ETH Zurich, Zurich, Switzerland.

^4^ Institute for Life Sciences, Department of Plant and Environmental Sciences, Hebrew University of Jerusalem, Jerusalem, Israel

^5^ Department of Plant and Environmental Sciences, Weizmann Institute of Science, Rehovot, Israel.

^6^ Institut de Ciències del Mar, CSIC, Barcelona, Catalonia, Spain

^7^ Max Planck Institute for Marine Microbiology, Bremen, Germany

*e-mail: [romanstocker@ethz.ch](mailto:romanstocker@ethz.ch) and [Jean-Baptiste.Raina@uts.edu.au](mailto:Jean-Baptiste.Raina@uts.edu.au)

**Supplementary Notes**

**Supplementary Note 1:**

To determine whether preferential chemotaxis to polymers (compared to oligomer or monomer constituents) is restricted to laminarin, we quantified the chemotactic responses of the four isolates towards alginate (another highly abundant marine polysaccharide^40^), to a mixture of its oligomers, and to the monomer mannuronate (1 mg mL^-1^ for all chemicals). Alginate is the main building block of brown algal cell walls and is a biofilm constituent in many bacteria^40,80^, often co-occurring with its derived oligomers and monomer. We observed strong attraction towards alginate, with an average chemotactic index of 11.9 ± 3.7 (Fig. S3a-d, ANOVA, *p* < 0.05 for the four strains in comparison with artificial seawater, Supplementary Data 15). This result demonstrates that strong bacterial chemotaxis towards large soluble polymers is not restricted to laminarin and may in fact be a common phenomenon across soluble polymers in the ocean. Similarly to laminarin, alginate elicited a chemotactic response that was on average 7.8 times larger than the mixture of oligomers, and 6.7 times larger than the monomer (Fig. S3, ANOVAs, *p* < 0.05 for all, Supplementary Data 15).

**Supplementary Note 2**:

DMSP_d_ in our laboratory experiments was only present in the surrounding seawater and was absent from the wells of the ISCA, and therefore could not induce chemotaxis into the ISCA wells. Nonetheless, we performed additional assays to test the ability of DMSP_d_ to induce chemotaxis in our four bacterial isolates, at the same concentration range used in our laboratory experiments (0.1-10 µM; Fig. S13). Among the four strains, we measured a chemotactic index of 2.45 ± 0.42 in *Alteromonas* sp. ASV109 to 0.1 µM DMSP_d_ (Fig. S13a, ANOVA, *p* < 0.05; Supplementary Data 29) and 1.77 ± 0.09 in *Pseudoalteromonas* sp. ASV39 to 1 µM DMSP_d_ (Fig. S13b, ANOVA, *p* < 0.05; Supplementary Data 29). *Pseudoalteromonas* sp. ASV16 and *Alteromonas* sp. ASV76 did not exhibit chemotaxis to any concentrations of DMSP_d_ (Fig. S13c,d, ANOVA, *p* > 0.05, Supplementary Data 29). We highlight again that, although two of the strains tested were weakly attracted to DMSP_d_, this compound was homogenously mixed in the surrounding seawater in our experiments measuring chemotaxis towards laminarin (Fig. 3c-f). Therefore, it could not attract cells into the ISCA wells.

**Supplementary Note 3:**

The increase in chemotaxis to laminarin upon amending the seawater outside of the ISCA wells (but not the wells themselves) with DMSP_d_ is not due to a potential repulsive effect from DMSP_d_ driving more cells away from the outside seawater into the wells. This can be seen from the fact that the number of cells in the control wells containing artificial seawater (without DMSP_d_) is the same whether DMSP_d_ has been added to the seawater outside the wells or not (Fig. S3c-f, ANOVA, *p* > 0.05 for all strains, Supplementary Data 30).

**Supplementary Note 4**:

In order to further elucidate whether the impact of DMSP on chemotaxis is limited to large polymers, we tested two additional small molecules -- the amino acids serine and aspartate -- with our four marine isolates in the presence of DMSP at different concentrations (Fig. S14a-h). No chemotactic response towards these two amino acids was measured and, additionally, no difference in attraction was recorded in the presence of DMSP (Fig. S14, ANOVA, *p* > 0.05; Supplementary Data 31). These results suggest that DMSP does not stimulate chemoattraction towards molecules that cells are not already attracted to. Further experiments to test the generality of the enhancing effect of DMSP were conducted using two other small compounds, spermidine and trimethylamine (TMA), as well as 10% Marine Broth 2216 (Fig. S15a), a rich medium containing a mixture of Peptone and yeast extract. While all three compounds induced a significant chemotactic response (Fig. S15a-c, ANOVA, *p* < 0.05; Supplementary Data 31), only spermidine induced a significantly greater response in the presence of 1 µM DMSP compared to the case without DMSP (Fig. S15b, ANOVA, *p* < 0.05; Supplementary Data 31). While limited to the set of compounds we tested, these data suggest that the enhancement of chemotaxis by DMSP may apply primarily or be strongest for polysaccharides. Ecologically, this would be relevant in helping bacteria climb the steeper gradients expected for larger molecules in view of their lower diffusivity.

**Supplementary Figures**


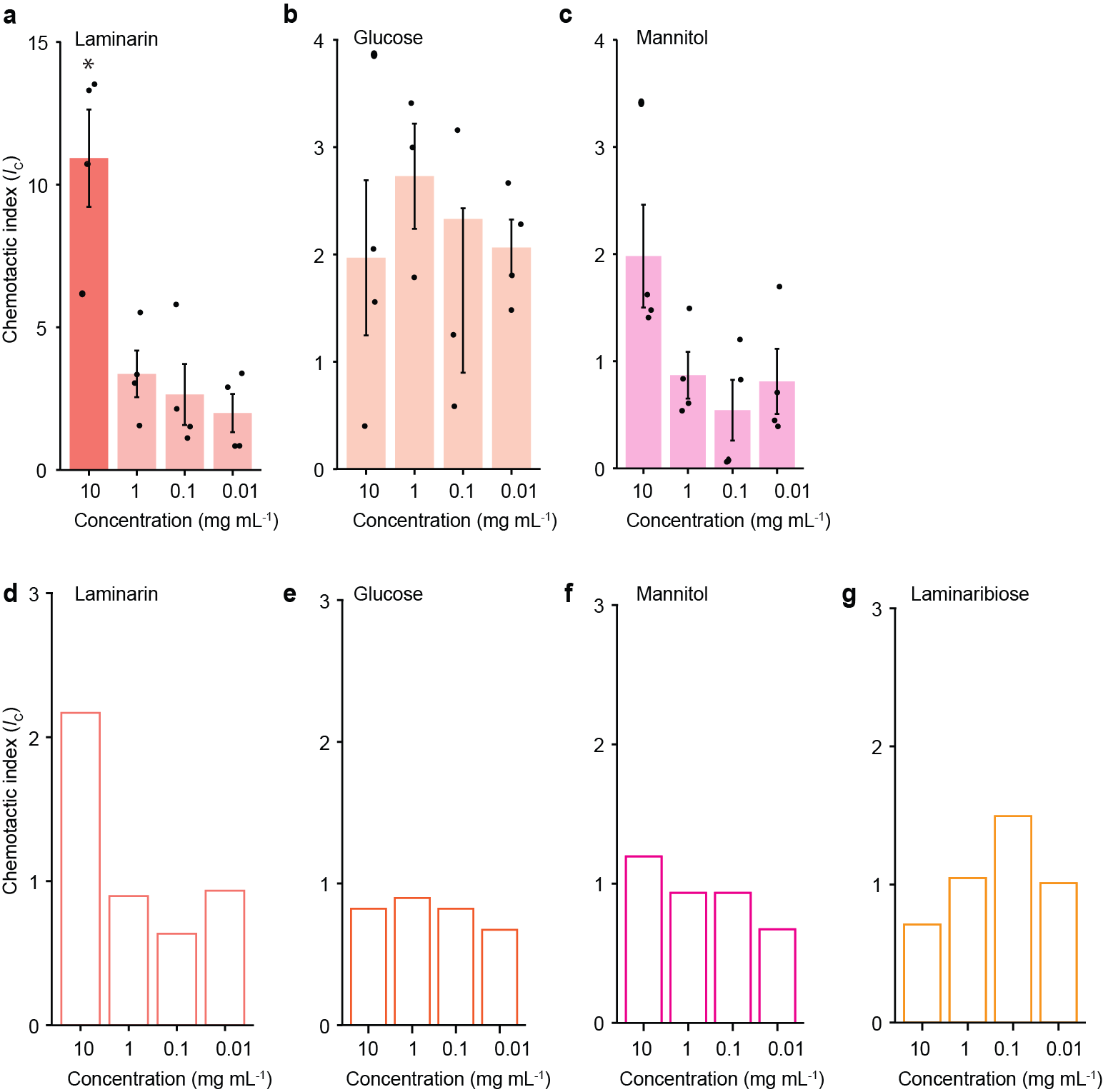


**Figure S1.** **Preliminary experiments to determine the optimal chemical concentrations to use in chemotaxis assays**. **(a-c)** Chemotactic index (*I*_C_, coloured bars, left axis) of *V. coralliilyticus* in response to a concentration range (10, 1, 0.1 and 0.01 mg mL^-1^) of laminarin (a), glucose (b), and mannitol (c) in laboratory conditions. An asterisk denotes a chemotactic index significantly larger than the artificial seawater controls (ANOVA, *p* < 0.05, Supplementary Data 1). Bars in darker shades indicate which concentrations elicited chemotactic responses significantly larger than the others (ANOVA, *p* < 0.05, Supplementary Data 1). Each treatment was replicated across four different ISCAs (*n* = 4 biologically independent experiments, denoted by individual dots). Bar plots represent the mean ± SD. **(d-g)** Chemotactic index (*I_C_*, coloured bars, left axis) of the fjord bacterial community towards a concentration range (10, 1, 0.1 and 0.01 mg mL^-1^) of laminarin (d), glucose (e), mannitol (f) and laminaribiose (g) *in situ*. This experiment was not replicated. Source data are provided as a Source Data file.


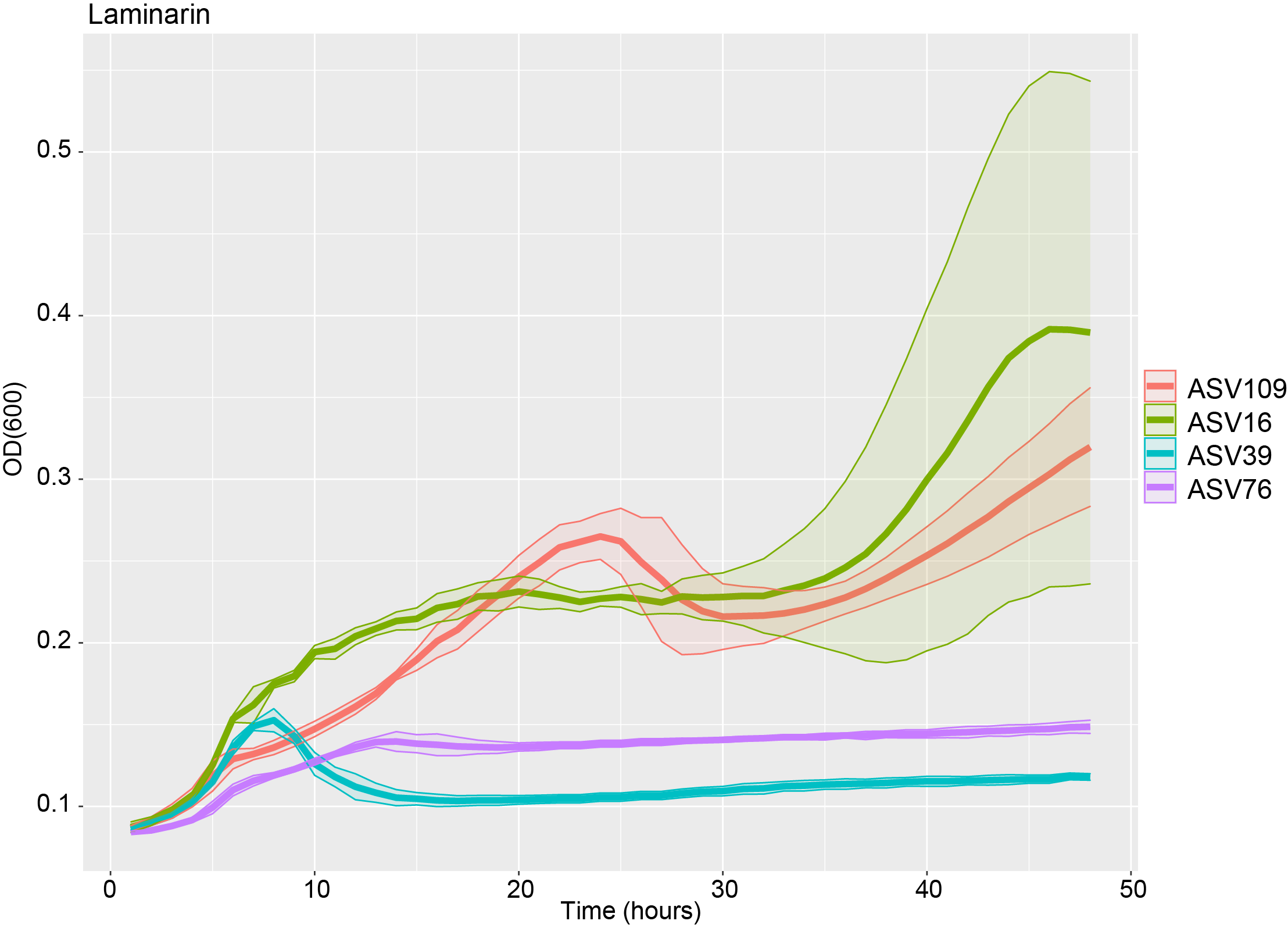


**Figure S2. Growth curves for ASV16 (green), ASV39 (blue), ASV76 (purple) and ASV109 (red) on 10 mg mL^-1^ laminarin, measured using a plate reader over 48 h.** From an overnight rich culture, cells were diluted 1:100 in artificial seawater containing 10 mg mL^-1^ laminarin, the highest concentration used in ISCA wells in our experiments. Experiments were performed in triplicate (n = 3 biologically independent measurement): thick solid lines represent the mean and shaded regions denote the standard deviation. Source data are provided as a Source Data file.


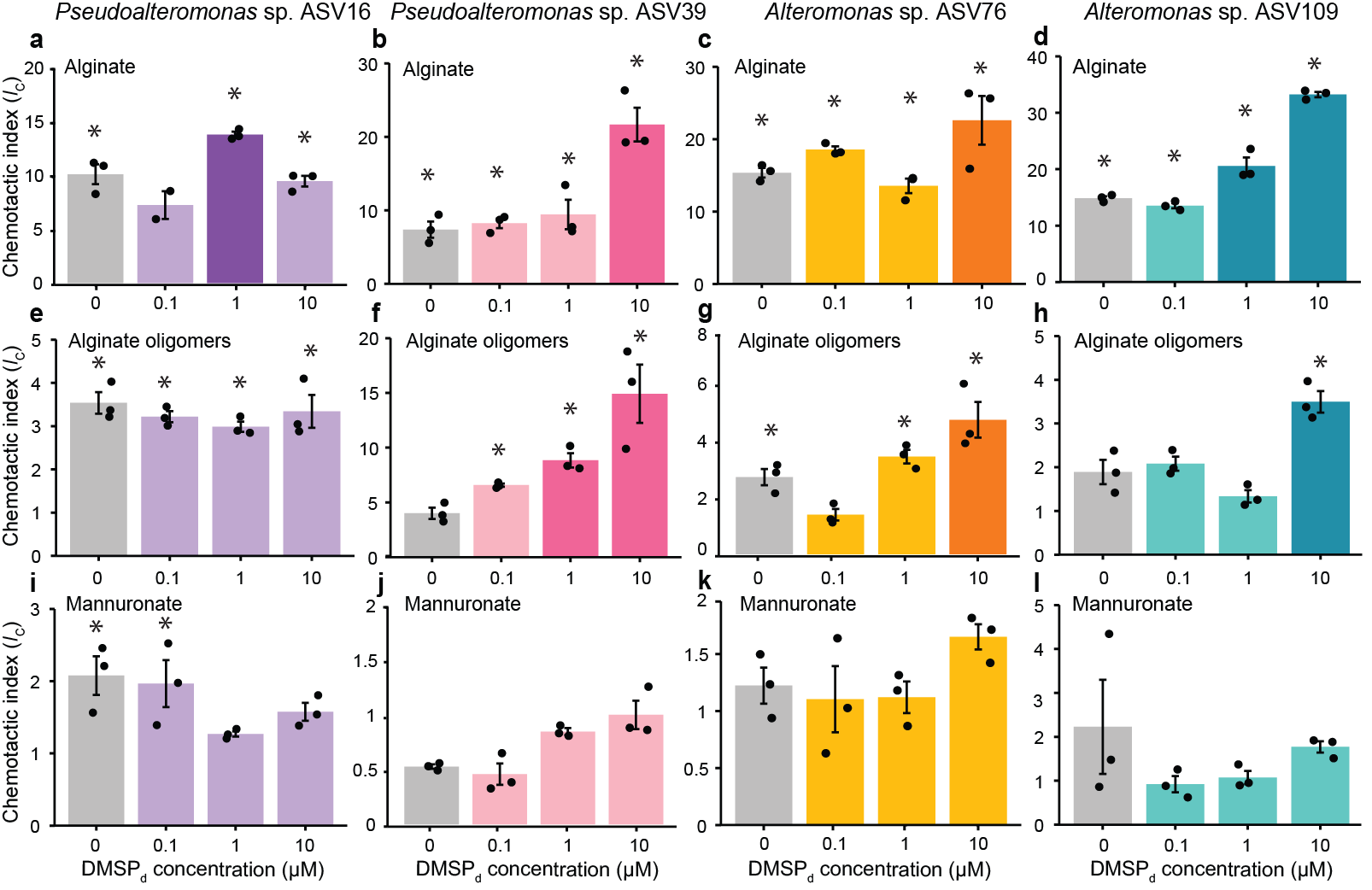


**Figure S3.** **Dissolved DMSP (DMSP_d_) significantly increases the strength of chemotaxis to alginate**. Chemotactic index (*I*_C_, coloured bars, left axis) in response to 1 mg mL^-1^ alginate (**a-d**), 1 mg mL^-1^ alginate oligomers (**e-h**) and 1 mg mL^-1^ mannuronate (**i-l**) in laboratory experiments in the presence of different concentrations of DMSP_d_ (0, 0.1 µM, 1 µM and 10 µM) in the surrounding seawater for *Pseudoalteromonas* sp*.* ASV16 (**a**,**e**,**i**), *Pseudoalteromonas* sp*.* ASV39 (**b**,**f**,**j**), *Alteromonas* sp. ASV76 (**c**,**g**,**k**), (e) and *Alteromonas* sp. ASV109 (**d**,**h**,**l**). An asterisk denotes a chemotactic index significantly larger than the artificial seawater controls (ANOVA, *p* < 0.05, Supplementary Data 20-21). Bars in darker shades indicate which DMSP_d_ concentrations elicited chemotactic responses that were significantly larger than the control without DMSP_d_ (ANOVA, *p* < 0.05, Supplementary Data 20-21). Each treatment was replicated across three different ISCAs (*n* = 3 biologically independent experiments, denoted by individual dots). Bar plots represent the mean ± SD. Source data are provided as a Source Data file.


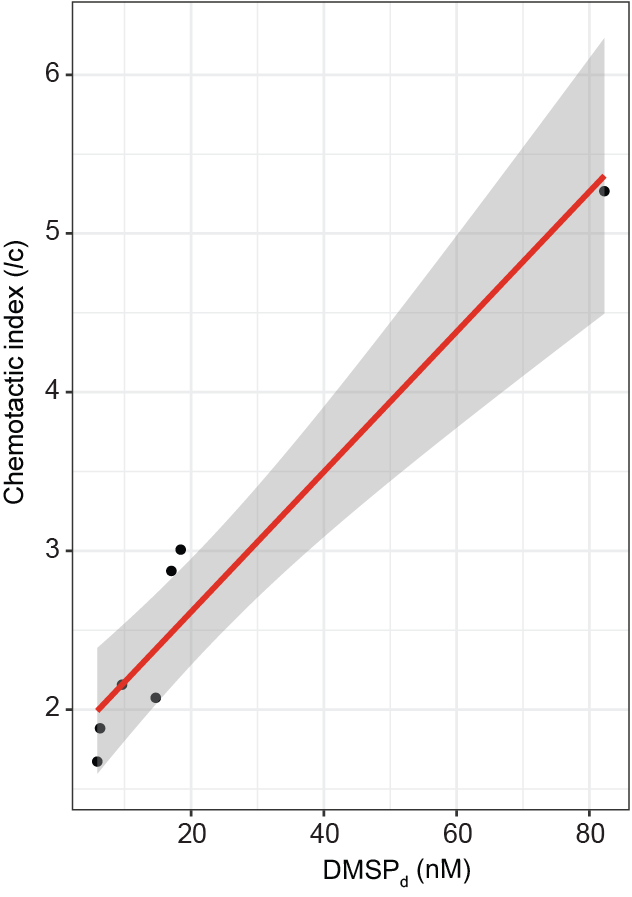


**Figure S4.** **Dissolved DMSP (DMSP_d_) concentration correlates significantly with the strength of chemotaxis to laminarin in field ISCA experiments**. The red line shows a linear regression fitted to the values of chemotactic index (*I*_C_, left axis) averaged per time point as a function of environmental DMSP_d_ concentration measurements (in nM, bottom axis), *R*^2^ = 0.97 (*F*-test (one-sided), *p* = 2.69 × 10^-5^).


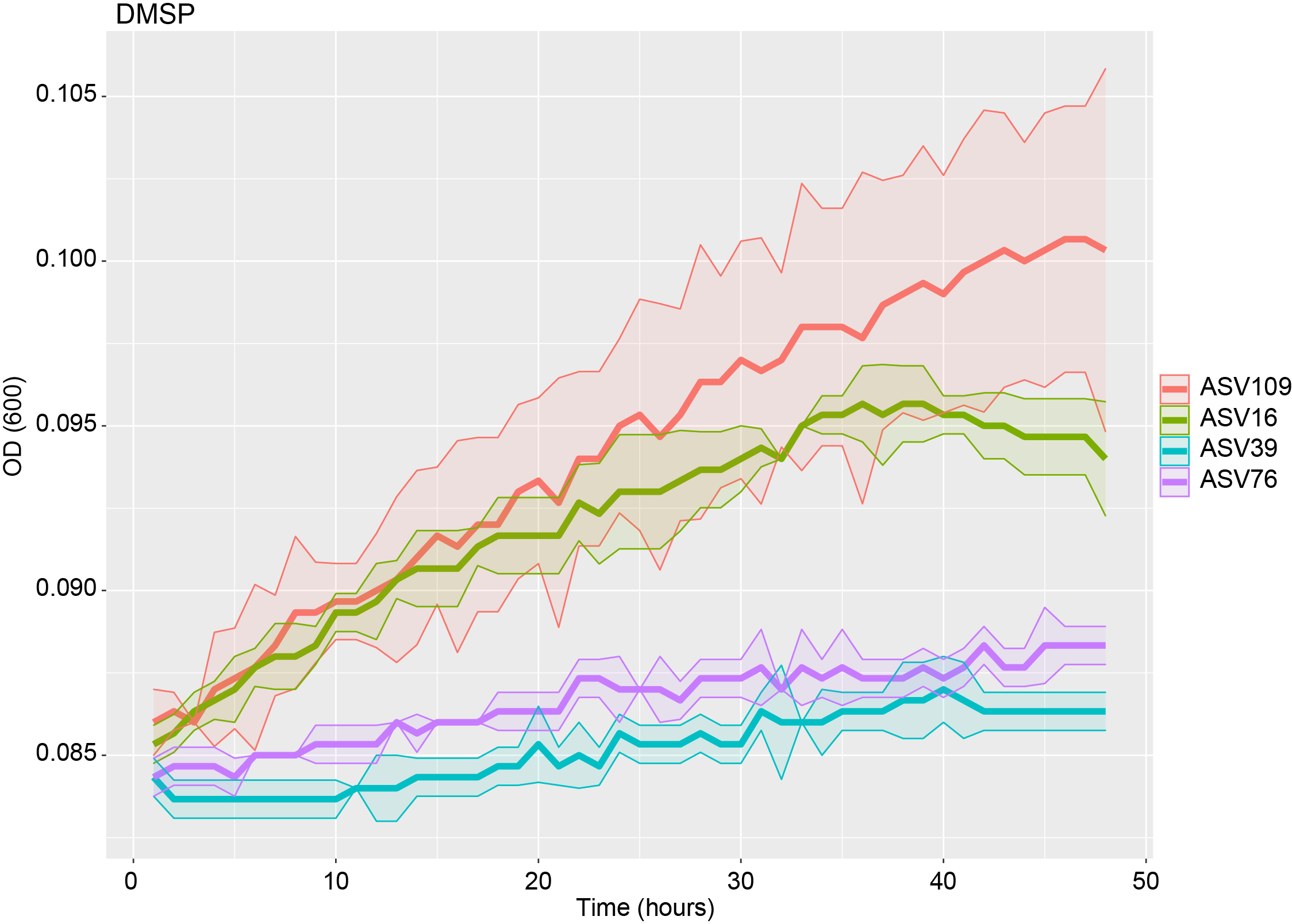


**Figure S5. Growth curves for ASV16 (green), ASV39 (blue), ASV76 (purple) and ASV109 (red) on 10 mM DMSP, measured using a plate reader over 48 h.** From an overnight rich culture, cells were diluted 1:100 in artificial seawater containing 10 mM DMSP to mimic the conditions outside ISCA wells in some of our experiments. Experiments were performed in triplicate (n = 3 biologically independent measurements): thick solid lines represent the mean and shaded regions denote the standard deviation. Source data are provided as a Source Data file.


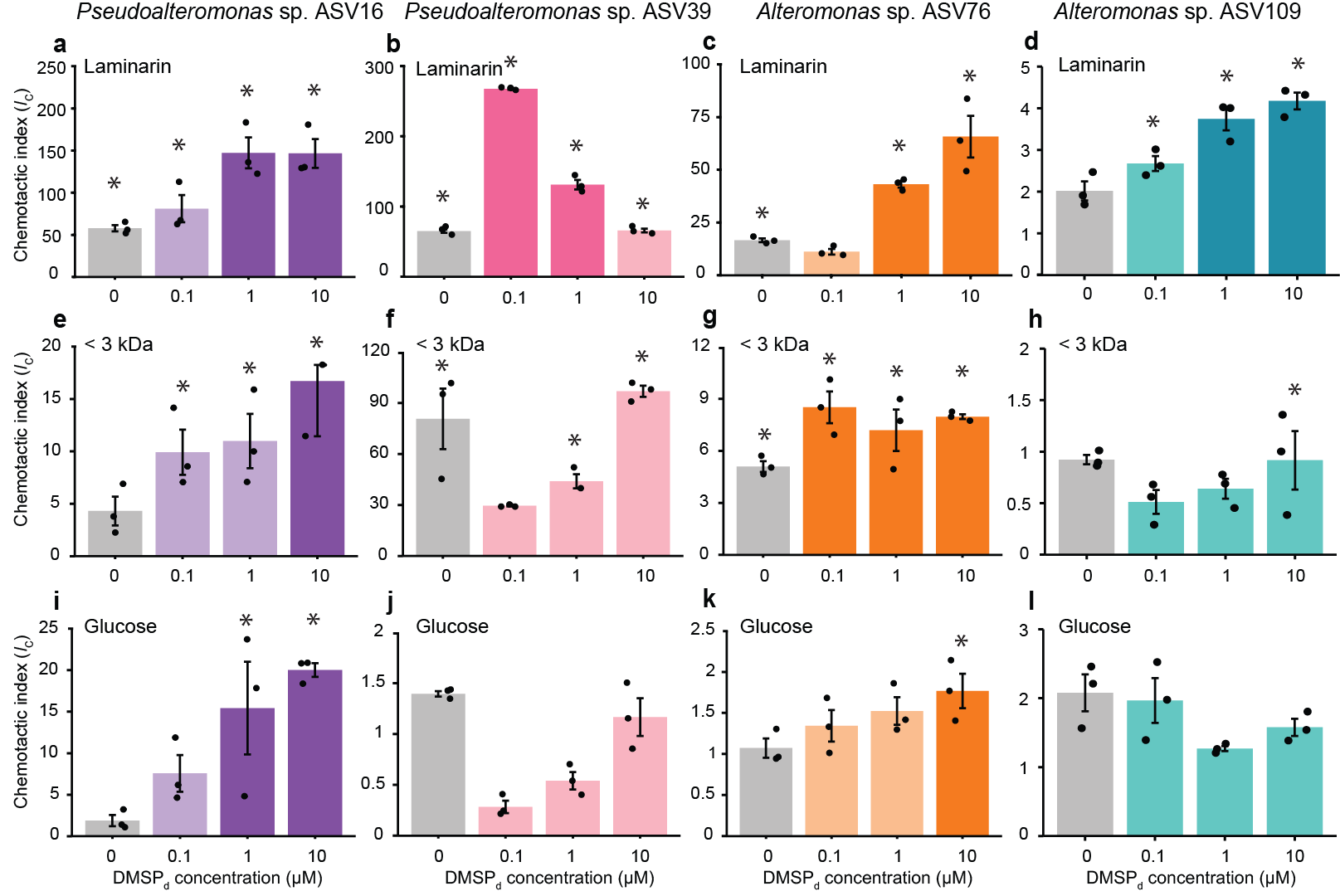


**Figure S6.** **Dissolved DMSP (DMSP_d_) significantly increases the strength of chemotaxis to laminarin**. Chemotactic index (*I*_C_, coloured bars, left axis) in response to 10 mg mL^-1^ laminarin (**a-d**), laminarin fraction (< 3 kDa) (**e-h**) and 1 mg mL^-1^ glucose (**i-l**) in laboratory experiments in the presence of different concentrations of DMSP_d_ (0, 0.1 µM, 1 µM and 10 µM) in the surrounding seawater for *Pseudoalteromonas* sp*.* ASV16 (**a**,**e**,**i**), *Pseudoalteromonas* sp*.* ASV39 (**b**,**f**,**j**), *Alteromonas* sp. ASV76 (**c**,**g**,**k**), (e) and *Alteromonas* sp. ASV109 (**d**,**h**,**l**). An asterisk denotes a chemotactic index significantly larger than the artificial seawater controls (ANOVA, *p* < 0.05, Supplementary Data 19). Bars in darker shades indicate which DMSP_d_ concentrations elicited chemotactic responses that were significantly larger than the control without DMSP_d_ (ANOVA, *p* < 0.05, Supplementary Data 19). Each treatment was replicated across three different ISCAs (*n* = 3 biologically independent experiments, denoted by individual dots). Bar plots represent the mean ± SD. Source data are provided as a Source Data file.


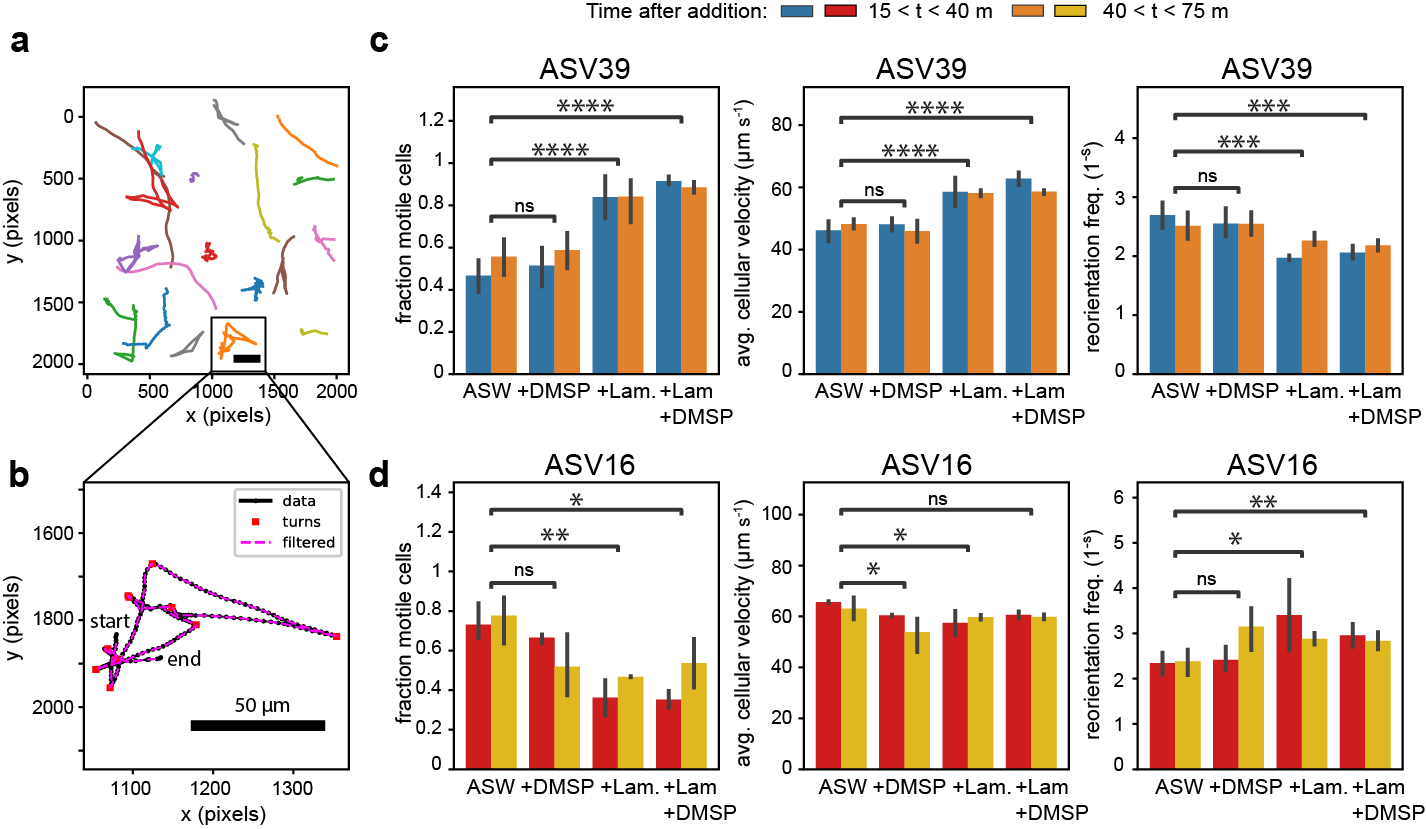


**Figure S7**. **Swimming speed and reorientation frequency of two marine isolates when exposed to DMSP_d_ and laminarin**. **(a)** Selected representative trajectories of the run-reverse-flick random motility of *Pseudoalteromonas* sp*.* ASV16 in artificial seawater (ASW), as obtained from cell tracking procedure (see Methods). Scale bar = 50 µm. **(b)** Detected reorientation events in an example trajectory from panel (a). **(c)** Motility parameters obtained from tracking *Pseudoalteromonas* sp*.* ASV39 in artificial seawater (ASW) (*n*_total_ = 1.6 × 10^4^ cells) supplemented with 10 µM DMSP_d_ (*n* = 2.3 × 10^4^ cells) and/or 0.1% (w/v) laminarin (*n* = 1.6 × 10^4^ / 1.4 × 10^4^ cells). (Left) Fraction of motile cells (*v* >12 µm s^-1^) in the sample. (Middle) Average velocity (mm s^-1^) of all motile cells. (Right) Average reorientation frequency per cell (1^-s^). Error bars indicate 95% confidence intervals. Mann-Whitney statistical test results indicated between each pair. Ns: not significant. *: *p* < 0.05; **: *p* < 0.01; ***: *p* < 0.001: ****: *p* < 0.0001. **(d)** As in panel c), but then for *Pseudoalteromonas* sp*.* ASV16 (ASW, *n* = 4.2 × 10^3^; DMSP_d_, *n* = 2.4 × 10^3^; Laminarin, *n* = 7.3 × 10^3^; both, *n* = 7.8 × 10^3^ cells).


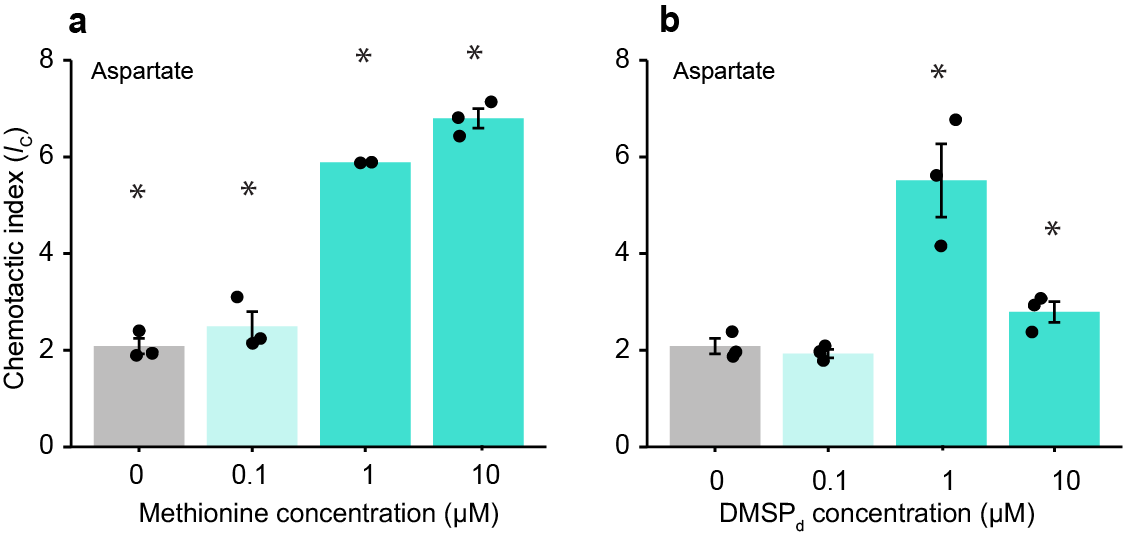


**Figure S8.** **Methionine and DMSP_d_ significantly increase the strength of chemotaxis to aspartate in *E. coli***. Chemotactic index (*I*_C_, coloured bars, left axis) of *E. coli* in response to 1 mM aspartate in laboratory experiments in the presence of different concentrations of **(a)** methionine and **(b)** DMSP_d_ (0, 0.1 µM, 1 µM and 10 µM for both molecules) in the surrounding seawater. The chemotactic response of *E. coli* in all methionine treatments and in 1 and 10 µM DMSP_d_ treatments was significantly larger than the artificial seawater controls (ANOVA, *p* < 0.05, Supplementary Data 22) and is indicated by an asterisk. Bars in darker shades indicate which methionine or DMSP_d_ concentrations elicited chemotactic responses that were significantly larger than the control without these compounds (ANOVA, *p* < 0.05, Supplementary Data 22). The chemotactic response induced by 1 mM methionine is not significantly different from 1 mM DMSP_d_ (ANOVA, *p* > 0.05, Supplementary Data 22). Each treatment was replicated across three different ISCAs (*n* = 3 biologically independent experiments, denoted by individual dots). Bar plots represent the mean ± SD. Source data are provided as a Source Data file.


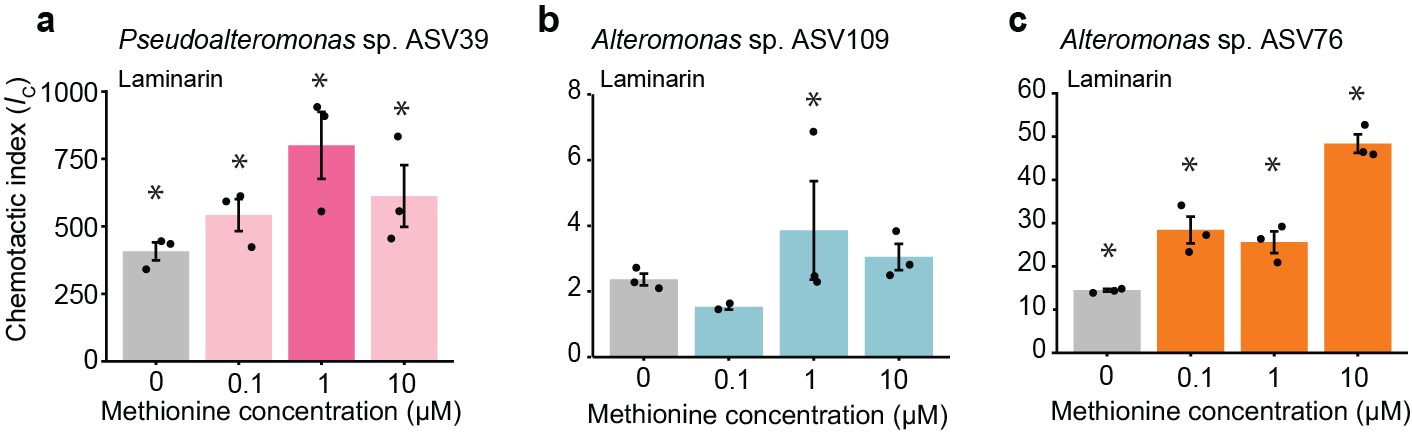


**Figure S9. Methionine significantly increases the strength of chemotaxis to laminarin.**  Chemotactic index (*I*_C_, coloured bars, left axis) in response to 10 mg mL^-1^ laminarin in laboratory experiments in the presence of different concentrations of methionine (0, 0.1 µM, 1 µM and 10 µM) in the surrounding seawater for the environmental isolates *Pseudoalteromonas* sp. ASV39 **(a)**, *Alteromonas* sp. ASV109 **(b)** and *Alteromonas* sp. ASV76 **(c)**. Note: the response of *Pseudoalteromonas* sp. ASV16 is reported in Figure 4. An asterisk denotes a chemotactic index significantly larger than the artificial seawater controls (ANOVA, *p* < 0.05, Supplementary Data 23). Bars in darker shades indicate which methionine concentrations elicited chemotactic responses that were significantly larger than the control without methionine (ANOVA, *p* < 0.05, Supplementary Data 23). Each treatment was replicated across three different ISCAs (*n* = 3 biologically independent experiments, denoted by individual dots). Bar plots represent the mean ± SD. Source data are provided as a Source Data file.


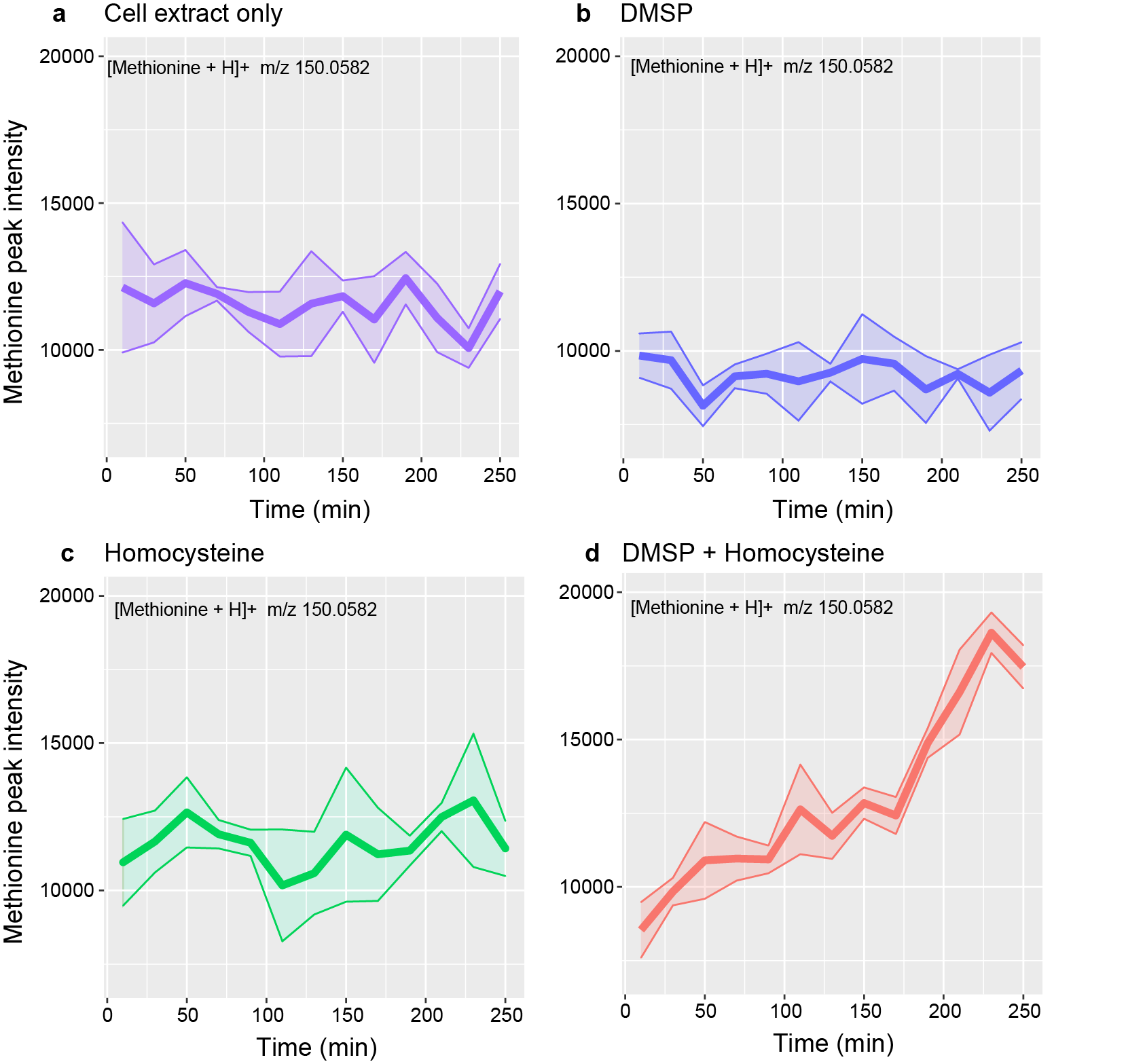


**Figure S10. Production of methionine by *Pseudoalteromonas* sp. ASV16 cell lysate in an *in vitro* enzyme assay**. Methionine peak intensity measured using mass spectrometry ([Methionine + H]+ m/z 150.0582) for the cell lysate when untreated (a), supplemented with 200 µM of DMSP (b), 200 µM of homocysteine (c) and 200 µM of both DMSP and homocysteine (d). Each treatment was sampled continuously in triplicate (n = 3 biologically independent measurements), with line plots representing mean (thick line) ± SD (shaded region).


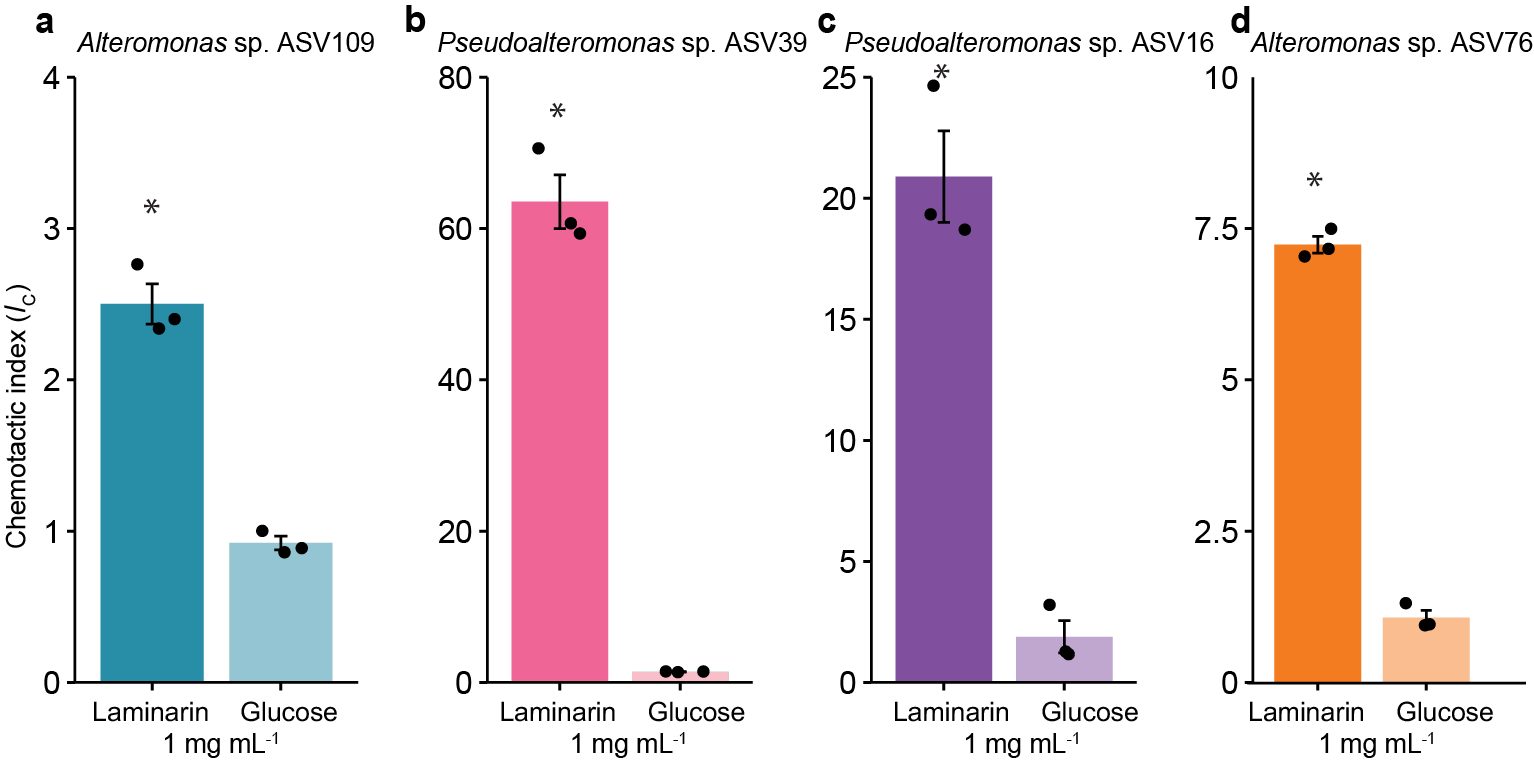


**Figure S11.** **The effect of a lower laminarin concentration on the chemotactic response of the four marine isolates.** Chemotactic index (*I*_C_, coloured bars, left axis) in response to 1 mg mL^-1^ laminarin and 1 mg mL^-1^ glucose in laboratory experiments for the four environmental isolates *Alteromonas* sp. ASV109 **(a)**, *Pseudoalteromonas* sp. ASV39 **(b)** *Pseudoalteromonas* sp. ASV16 **(c)**, and *Alteromonas* sp. ASV76 **(d)**. An asterisk denotes a chemotactic index significantly larger than the artificial seawater controls (ANOVA, *p* < 0.05, Supplementary Data 27). Bars in darker shades indicate that the chemotactic response to laminarin is significantly stronger than glucose (ANOVA, *p* < 0.05, Supplementary Data 27). Each treatment was replicated across three different ISCAs (*n* = 3 biologically independent experiments, denoted by individual dots). Bar plots represent the mean ± SD. Source data are provided as a Source Data file.


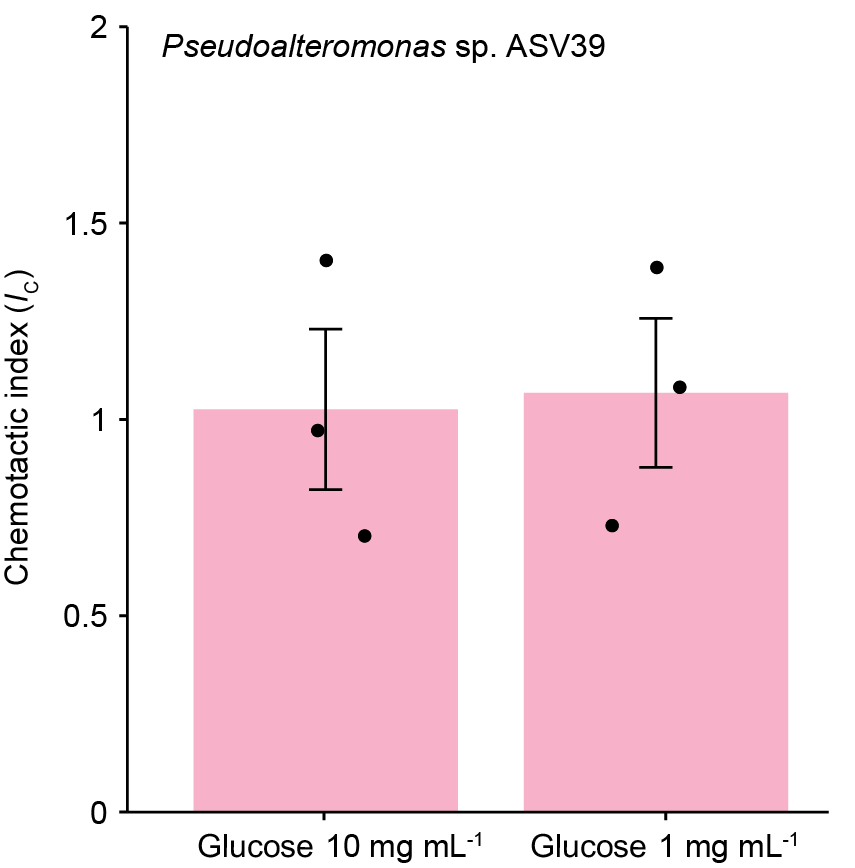


**Figure S12.** **The effect of glucose concentrations on the chemotactic response of *Pseudoalteromonas* sp. ASV39.** Chemotactic index (*I*_C_, coloured bars, left axis) in response to 10 mg mL^-1^ glucose and 1 mg mL^-1^ glucose in laboratory experiments for *Pseudoalteromonas* sp. ASV39. The chemotactic responses were not significantly higher than the artificial seawater controls (ANOVA, *p* > 0.05 for both, Supplementary Data 28), nor different between the two concentrations (ANOVA, *p* > 0.05, Supplementary Data 28). Each treatment was replicated across three different ISCAs (*n* = 3 biologically independent experiments, denoted by individual dots). Bar plots represent the mean ± SD. Source data are provided as a Source Data file.


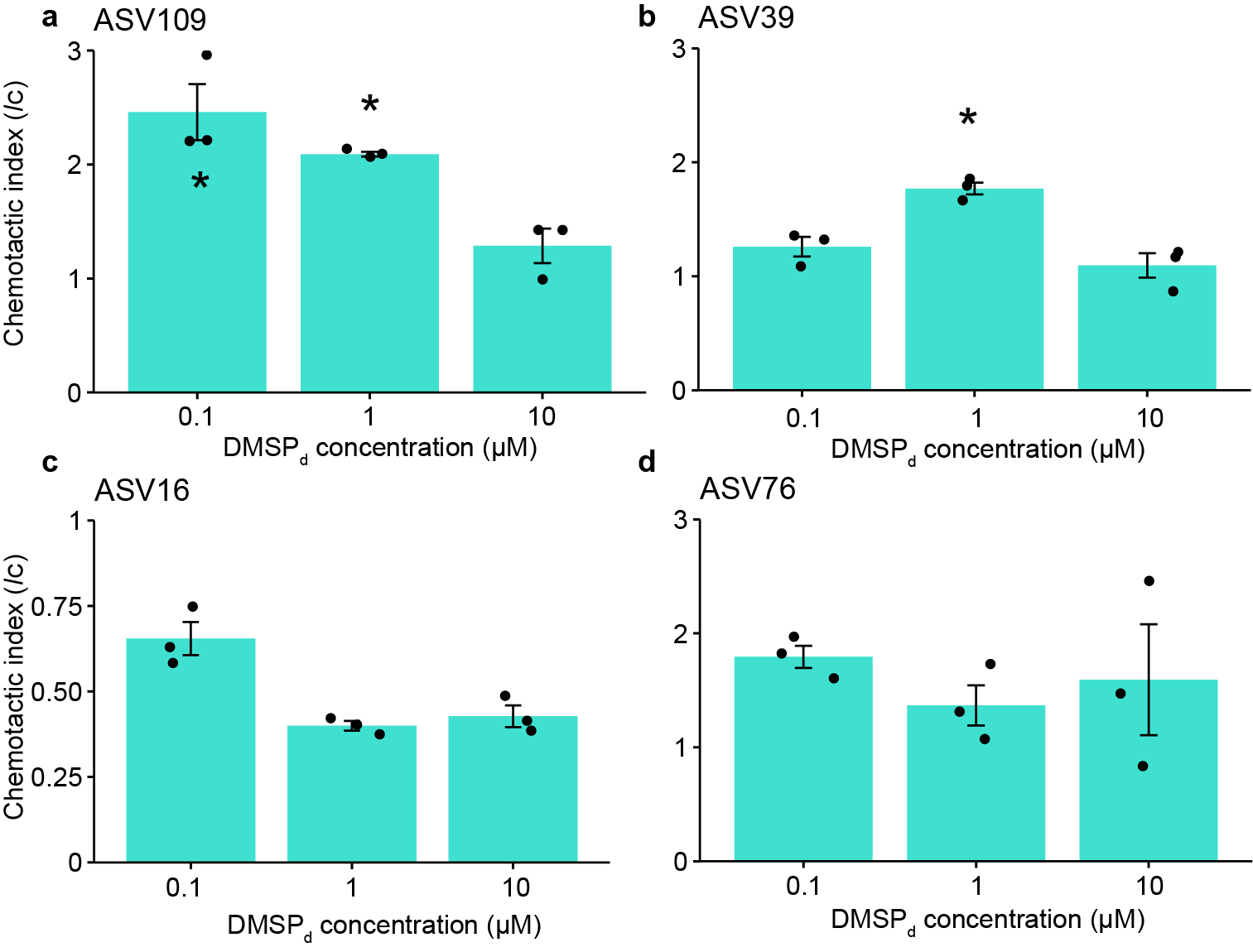


**Figure S13. Chemotactic response of the four marine strains to DMSP_d_ at different concentrations.** Chemotactic index (*I*_C_, coloured bars) of *Alteromonas* sp. ASV109 (a), *Pseudoalteromonas* sp. ASV39 (b), *Pseudoalteromonas* sp. ASV16 (c) and *Alteromonas* sp. ASV76 (d), in response to 0.1, 1 and 10 µM of DMSP_d_ in laboratory ISCA experiments. An asterisk denotes positive chemotaxis, i.e. a chemotactic index significantly larger than 1 (ANOVA, *p* < 0.05; Supplementary Data 29). Each treatment was replicated across three different ISCAs (*n* = 3 biologically independent experiments). Bar plots represent the mean (colored bar) ± SD (error bar), with replicates shown as individual dots. Source data are provided as a Source Data file.


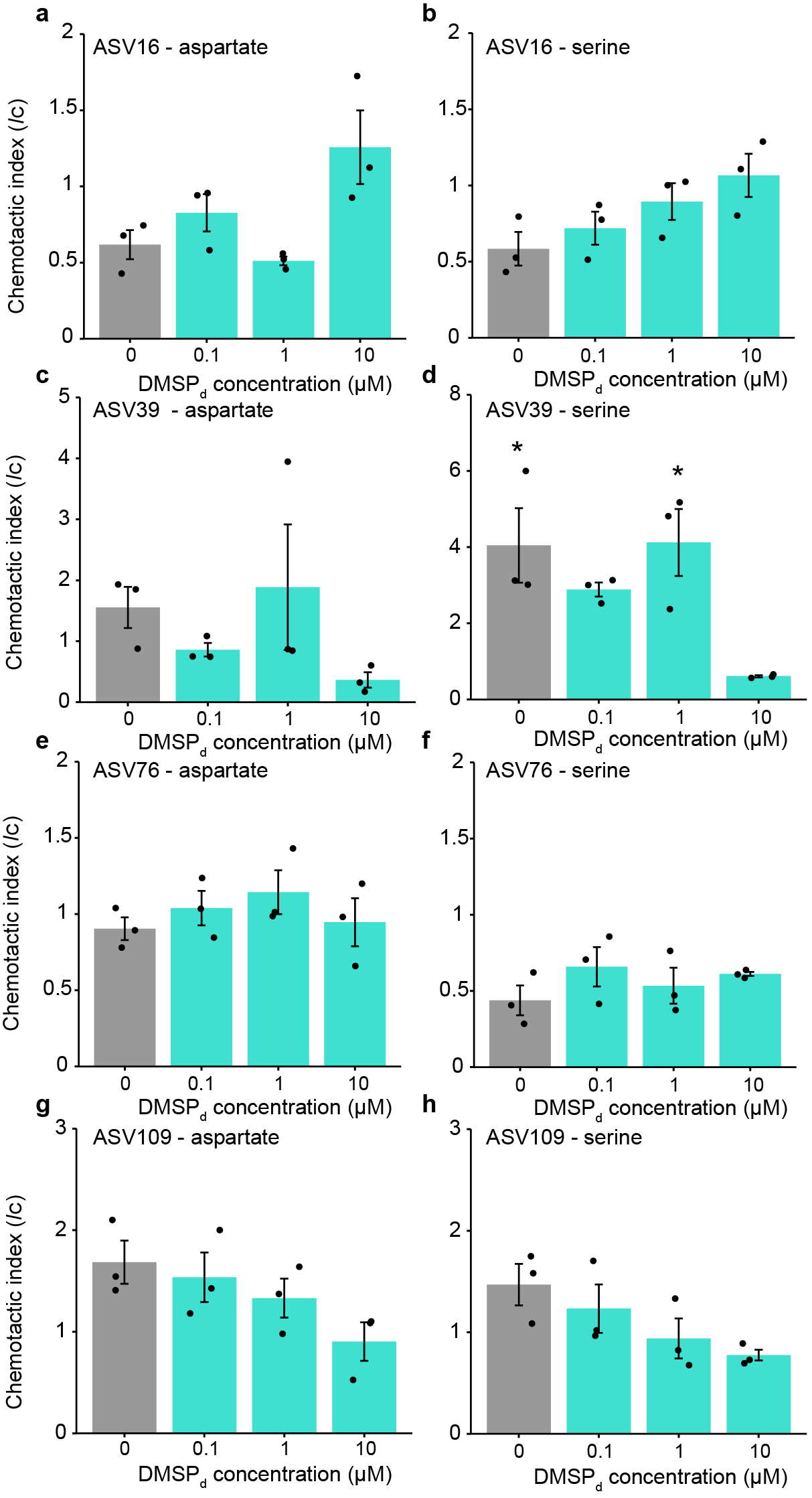


**Figure S14: Chemotactic index (*I*_C_, coloured bars) in response to 1 mM aspartate (a,c,e,g) and 1 mM serine (b,d,f,h) in ISCA laboratory experiments, in the presence and absence of DMSP****_d_ at different concentrations.** Experiments were performed without DMSP_d_ (grey bars) and in the presence of three different concentrations of DMSP_d_ (0, 0.1, 1 and 10 µM; cyan bars) in the surrounding artificial seawater, for *Pseudoalteromonas* sp. ASV16 (a-b), *Alteromonas* sp. ASV39 (c-d), *Pseudoalteromonas* sp. ASV76 (e-f) and *Alteromonas* sp. ASV109 (g-h). In no case was the chemotactic response significantly larger in the presence of DMSP_d_ compared to the absence of DMSP_d_ (ANOVA, *p* > 0.05, Supplementary Data 31). An asterisk denotes positive chemotaxis, i.e., a chemotactic index significantly larger than 1 (ANOVA, *p* < 0.05; Supplementary Data 31). Each treatment was replicated across three different ISCAs (*n* = 3 biologically independent experiments). Bar plots represent the mean (colored bar) ± SD (error bar), with replicates shown as individual dots. Source data are provided as a Source Data file.


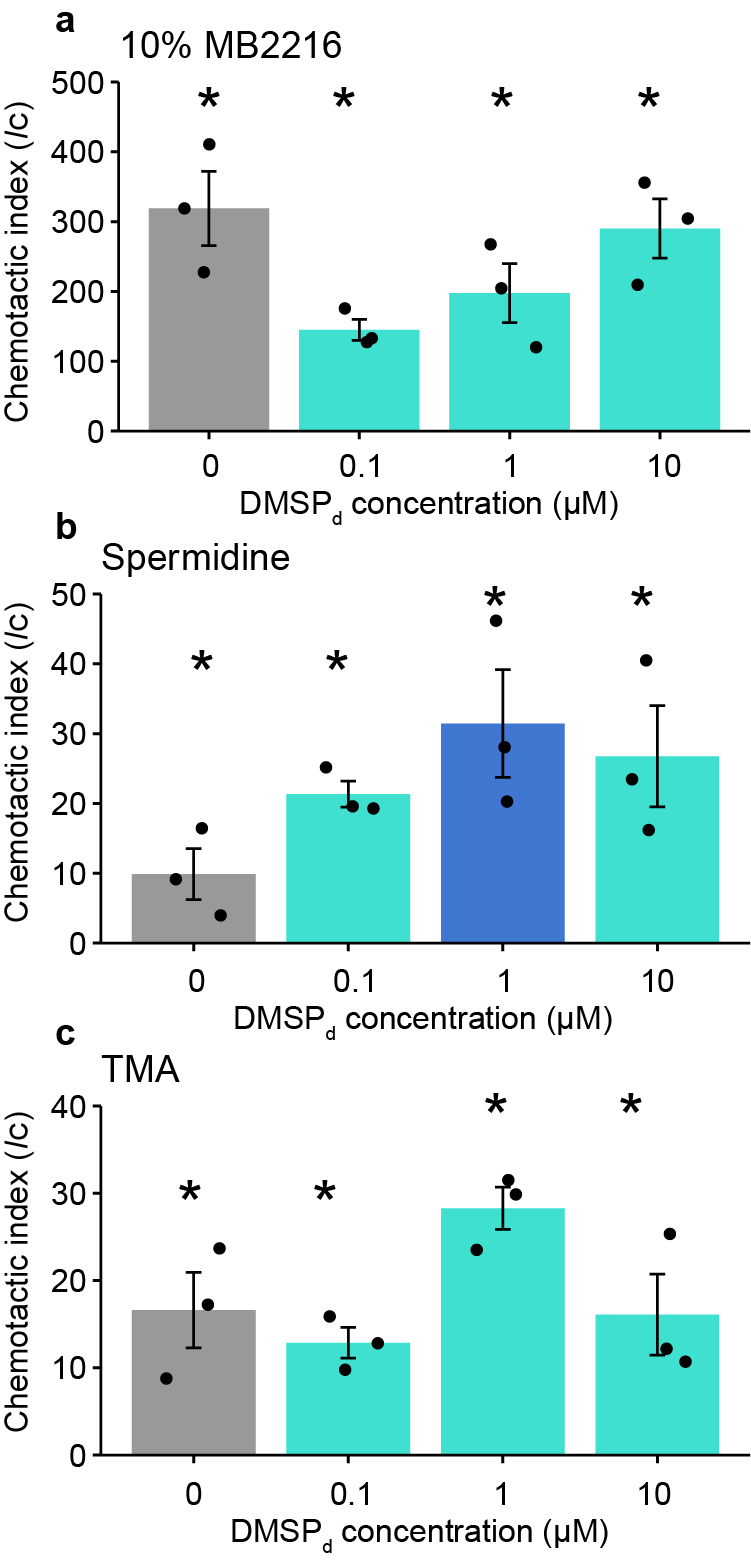


**Figure S15. Chemotactic response of *Pseudoalteromonas* sp. ASV16 to 10% Marine Broth and two small compounds in the presence of different concentrations of DMSP_d_.** Chemotactic index (*I*_C_, coloured bars) of *Pseudoalteromonas* ASV16 in response to 10% Marine Broth 2216 (a), spermidine (b) and trimethylamine (TMA, c), in the presence of different concentrations of DMSP (0, 0.1, 1 and 10 µM) in the surrounding artificial seawater. An asterisk denotes a chemotactic index significantly larger than 1 (ANOVA, *p* < 0.05; Supplementary Data 31). The bar in darker shade indicates that the chemotactic response to spermidine was significantly larger than the treatment without DMSP_d_ (ANOVA, *p* < 0.05; Supplementary Data 31). Each treatment was replicated across three different ISCAs (*n* = 3 biologically independent experiments). Bar plots represent the mean (colored bar) ± SD (error bar), with replicates shown as individual dots. Source data are provided as a Source Data file.


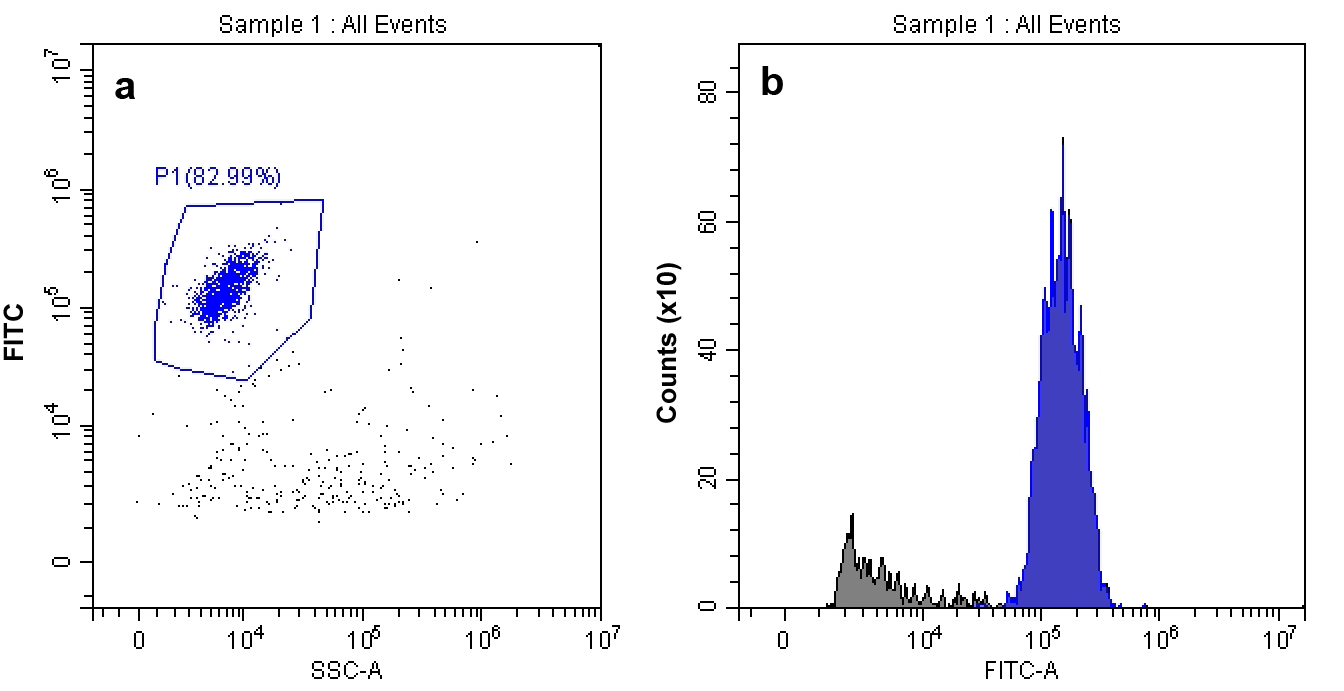


**Figure S16. Gating strategy used to collect flow cytometry data**. (a) Prokaryotic populations were characterized according to side scatter (SSC) and SYBR Green fluorescence (FITC). (b) Counts enumerating the cells in the prokaryotic gate.
