# Supplementary material for: Strong chemotaxis by marine bacteria towards polysaccharides is enhanced by the abundant organosulfur compound DMSP
Source: Nat Commun. 2023 Dec 6;14:8080. doi: 10.1038/s41467-023-43143-z (PMC10700628; doi:10.1038/s41467-023-43143-z)
Supplement: Supplementary file 3 — Description of Additional Supplementary Files [file 41467_2023_43143_MOESM3_ESM.pdf]

Title: Supplementary Data 1:
Description: Sums of squares (SS), mean squares (MS) and significance levels for the analyses of variance (ANOVAs, one-sided) of the chemotactic indexes of V. coralliilyticus reported in Fig. S1a-c (diff: differences in mean; lower and upper: confidence intervals). FSW: Filtered seawater negative control.

Title: Supplementary Data 2:
Description: Sums of squares (SS), mean squares (MS) and significance levels for the analyses of variance (ANOVAs, one-sided) of the chemotactic responses reported in Fig. 1ab (diff: differences in mean; lower and upper: confidence intervals). FSW: seawater negative control.

Title: Supplementary Data 3:
Description: Summary of permutational multivariate analysis of variance (PERMANOVA) comparing the taxonomic composition of the prokaryotic communities between laminarin samples and surrounding seawater (Fig. 1c). Bray-Curtis similarity, 999 unrestricted permutations, main effect and pairwise tests.

Title: Supplementary Data 4:
Description: Summary of permutational multivariate analysis of variance (PERMANOVA) comparing the taxonomic composition of the prokaryotic communities between laminarin samples and filtered seawater controls (Fig 1c). Bray-Curtis similarity, 999 unrestricted permutations, main effect and pairwise tests.

Title: Supplementary Data 5:
Description: Summary of the taxa-specific enrichments on day 3 between surrounding seawater (bulk) and laminarin samples using the R package ANCOM-BC. ANCOM-BC performs differential abundance (DA) analysis implementing Bonferroni correction to adjust for multiple comparisons.

Title: Supplementary Data 6:
Description: Summary of the taxa-specific enrichments on day 9 between surrounding seawater (bulk) and laminarin samples using the R package ANCOM-BC. ANCOM-BC performs differential abundance (DA) analysis implementing Bonferroni correction to adjust for multiple comparisons.

Title: Supplementary Data 7:
Description: Summary of the taxa-specific enrichments on day 15 between surrounding seawater (bulk) and laminarin samples using the R package ANCOM-BC. ANCOM-BC performs differential abundance (DA) analysis implementing Bonferroni correction to adjust for multiple comparisons.

Title: Supplementary Data 8:
Description: Summary of the taxa-specific enrichments on day 18 between surrounding seawater

(bulk) and laminarin samples using the R package ANCOM-BC. ANCOM-BC performs differential abundance (DA) analysis implementing Bonferroni correction to adjust for multiple comparisons.

Title: Supplementary Data 9:
Description: Summary of the taxa-specific enrichments on day 22 between surrounding seawater (bulk) and laminarin samples using the R package ANCOM-BC. ANCOM-BC performs differential abundance (DA) analysis implementing Bonferroni correction to adjust for multiple comparisons.

Title: Supplementary Data 10:
Description: Summary of the taxa-specific enrichments over all time points between surrounding seawater (bulk) and laminarin samples using the R package ANCOM-BC. ANCOM-BC performs differential abundance (DA) analysis implementing Bonferroni correction to adjust for multiple comparisons.

Title: Supplementary Data 11:
Description: Sequences alignment BLASTn results between 16S rRNA gene sequence of isolated strains on laminarin with enriched ASVs found in laminarin samples (% ID: percentage of identity between sequences).

Title: Supplementary Data 12:
Description: Sums of squares (SS), mean squares (MS) and significance levels for the analyses of variance (ANOVAs, one-sided) of the chemotactic responses reported in Fig. 2a-d (diff: differences in mean; lower and upper: confidence intervals). FSW: Filtered seawater negative control.

Title: Supplementary Data 13:
Description: Sums of squares (SS), mean squares (MS) and significance levels for the analyses of variance (ANOVAs, one-sided) of the growth rates calculated form the growth curves reported in Fig. 2e-h (diff: differences in mean; lower and upper: confidence intervals). FSW: Filtered seawater negative control.

Title: Supplementary Data 14:
Description: Average growth rate and duplication time of the four isolates grown on laminarin (10 mg mL-1) for 48h, displayed in Fig. S2e-h. The cells were prepared in the same growth conditions as for ISCA experiments.

Title: Supplementary Data 15:
Description: Sums of squares (SS), mean squares (MS) and significance levels for the analyses of variance (ANOVAs, one-sided) of the DMSP - free chemotactic indexes reported in Fig. S3 (diff: differences in mean; lower and upper: confidence intervals). FSW: Filtered seawater negative control.

Title: Supplementary Data 16:
Description: Correlation factor and p-value (Spearman) of the chemotactic responses measured throughout the bloom with the measurements of 61 environmental variables displayed in Fig. 3a.

Title: Supplementary Data 17:
Description: Concentrations of DMSPd (nM) in the surrounding seawater throughout the phytoplankton bloom displayed in Fig. 3b.

Title: Supplementary Data 18:
Description: Duplication time of the four isolates when grown on DMSP (10 mM) for 48h, displayed in Fig. S5. The cells were prepared in the same growth conditions as for ISCA experiments.

Title: Supplementary Data 19:
Description: Sums of squares (SS), mean squares (MS) and significance levels for the analyses of variance (ANOVAs, one-sided) of the chemotactic indexes reported in Fig. 3c-f and S6 (diff: differences in mean; lower and upper: confidence intervals). FSW: Filtered seawater negative control. 0: DMSP-free control; 0.1,1 and 10 corresponds to the concentration (µM) of DMSP added to the surrounding seawater.

Title: Supplementary Data 20:
Description: Sums of squares (SS), mean squares (MS) and significance levels for the analyses of variance (ANOVAs, one-sided) of the chemotactic indexes reported towards alginate in presence of DMSP in Fig. S3a-d (diff: differences in mean; lower and upper: confidence intervals). FSW: Filtered seawater negative control. 0: DMSP-free control; 0.1,1 and 10 corresponds to the concentration (µM) of DMSP added to the surrounding seawater.

Title: Supplementary Data 21:
Description: Sums of squares (SS), mean squares (MS) and significance levels for the analyses of variance (ANOVAs, one-sided) of the chemotactic indexes reported towards alginate oligomers and monomer in presence of DMSP in Fig. S3e-l (diff: differences in mean; lower and upper: confidence intervals). FSW: Filtered seawater negative control. 0: DMSP-free control; 0.1,1 and 10 corresponds to the concentration (µM) of DMSP added to the surrounding seawater.

Title: Supplementary Data 22:
Description: Sums of squares (SS), mean squares (MS) and significance levels for the analyses of variance (ANOVAs, one-sided) of the chemotactic indexes of E. coli reported towards 1 mM aspartate in presence of methionine or DMSP in Fig. S8 (diff: differences in mean; lower and upper: confidence intervals). Control: Filtered medium negative control. 0: DMSP or methionine-free control; 0.1,1 and 10 corresponds to the concentration (µM) of DMSP or methionine added to the

surrounding seawater. A last test compares the levels of chemotaxis by E. coli to aspartate in methionine vs DMSP.

Title: Supplementary Data 23:
Description: Sums of squares (SS), mean squares (MS) and significance levels for the analyses of variance (ANOVAs, one-sided) of the chemotactic indexes of the four marine isolates reported towards laminarin in presence of methionine in Fig. 4b and Fig. S9 (diff: differences in mean; lower and upper: confidence intervals). FSW: Filtered seawater negative control. 0: methionine-free control; 0.1,1 and 10 corresponds to the concentration (µM) of methionine added to the surrounding seawater.

Title: Supplementary Data 24:
Description: Sums of squares (SS), mean squares (MS) and significance levels for the analyses of variance (ANOVAs, one-sided) of the chemotactic indexes of marine isolate Pseudoalteromonas sp. ASV16 reported towards laminarin in presence of choline in Fig. 4c (diff: differences in mean; lower and upper: confidence intervals). FSW: Filtered seawater negative control. 0: choline-free control; 0.1,1 and 10 corresponds to the concentration (µM) of choline added to the surrounding seawater.

Title: Supplementary Data 25:
Description: Sums of squares (SS), mean squares (MS) and significance levels for the analyses of variance (ANOVAs, one-sided) of the chemotactic indexes of marine isolate Pseudoalteromonas sp. ASV16 reported towards laminarin in presence of homocysteine in Fig. 4d (diff: differences in mean; lower and upper: confidence intervals). FSW: Filtered seawater negative control. 0: homocysteine-free control; 0.1,1 and 10 corresponds to the concentration (µM) of homocysteine added to the surrounding seawater.

Title: Supplementary Data 26:
Description: Closest sequenced bacterial relatives of the four marine strains (a) and DMSP catabolism genes orthology analysis (b).

Title: Supplementary Data 27:
Description: Sums of squares (SS), mean squares (MS) and significance levels for the analyses of variance (ANOVAs, one-sided) of the chemotactic indexes of the four isolates reported in Fig. S11 (diff: differences in mean; lower and upper: confidence intervals). FSW: Filtered seawater negative control.

Title: Supplementary Data 28:
Description: Sums of squares (SS), mean squares (MS) and significance levels for the analyses of variance (ANOVAs, one-sided) of the chemotactic indexes of isolate Pseudoalteromonas sp. ASV39 reported in Fig. S12 (diff: differences in mean; lower and upper: confidence intervals). FSW: Filtered seawater negative control.

Title: Supplementary Data 29:
Description: Sums of squares (Sum Sq), mean squares (Mean Sq) and significance levels for the analyses of variance (ANOVAs, one-sided) of the cell counts of the four isolates measured in different concentrations of DMSP and seawater negative control reported in Supplementary Note 2 and Fig. S13 (diff: differences in mean; lower and upper: confidence intervals). Data are reported per DMSP concentration (0.1, 1 and 10 µM) in the ISCA. FSW: Filtered seawater negative control.

Title: Supplementary Data 30:
Description: Sums of squares (Sum Sq), mean squares (Mean Sq) and significance levels for the analyses of variance (ANOVAs, one-sided) of the cell counts of the four isolates measured in the control wells containing artificial seawater reported in Supplementary Note 3 (diff: differences in mean; lower and upper: confidence intervals). Data are reported per DMSP concentration (µM) amended in the surrounding seawater - 0: DMSP-free control; 0.1,1 and 10 µM DMSP.

Title: Supplementary Data 31:
Description: Sums of squares (Sum Sq), mean squares (Mean Sq) and significance levels for the analyses of variance (ANOVAs, one-sided) of the cell counts of the isolates measured to serine (1 mM; a), aspartate (1mM; b), TMA (1mM; c) , spermidine (1mM; d) and MB2216 (10%; e) in different concentrations of DMSP and seawater negative control reported in Figs. S14 -S15. TMA, spermidine and MB2216 were tested only with Pseudoalteromonas sp. ASV16. (diff: differences in mean; lower and upper: confidence intervals). 0: DMSP-free control; 0.1,1 and 10 corresponds to the concentration (µM) of DMSP added to the surrounding seawater. FSW: Filtered seawater negative control.
