# Supplementary material for: Strong chemotaxis by marine bacteria towards polysaccharides is enhanced by the abundant organosulfur compound DMSP
Source: Nat Commun. 2023 Dec 6;14:8080. doi: 10.1038/s41467-023-43143-z (PMC10700628; doi:10.1038/s41467-023-43143-z)
Supplement: Supplementary file 5 — Reporting Summary [file 41467_2023_43143_MOESM5_ESM.pdf]

nature portfolio

Corresponding author(s): Roman Stocker; Jean-Baptiste Raina

Last updated by author(s): Oct 20, 2023

Reporting Summary

Nature Portfolio wishes to improve the reproducibility of the work that we publish. This form provides structure for consistency and transparency in reporting. For further information on Nature Portfolio policies, see our Editorial Policies and the Editorial Policy Checklist.

Statistics

For all statistical analyses, confirm that the following items are present in the figure legend, table legend, main text, or Methods section.

n/a	Confirmed	
☐	☒	The exact sample size (*n*) for each experimental group/condition, given as a discrete number and unit of measurement
☐	☒	A statement on whether measurements were taken from distinct samples or whether the same sample was measured repeatedly
☐	☒	The statistical test(s) used AND whether they are one- or two-sided<br>*Only common tests should be described solely by name; describe more complex techniques in the Methods section.*
☐	☒	A description of all covariates tested
☐	☒	A description of any assumptions or corrections, such as tests of normality and adjustment for multiple comparisons
☐	☒	A full description of the statistical parameters including central tendency (e.g. means) or other basic estimates (e.g. regression coefficient) AND variation (e.g. standard deviation) or associated estimates of uncertainty (e.g. confidence intervals)
☐	☒	For null hypothesis testing, the test statistic (e.g. *F*, *t*, *r*) with confidence intervals, effect sizes, degrees of freedom and *P* value noted<br>*Give P values as exact values whenever suitable.*
☒	☐	For Bayesian analysis, information on the choice of priors and Markov chain Monte Carlo settings
☒	☐	For hierarchical and complex designs, identification of the appropriate level for tests and full reporting of outcomes
☐	☒	Estimates of effect sizes (e.g. Cohen's *d*, Pearson's *r*), indicating how they were calculated

Our web collection on statistics for biologists contains articles on many of the points above.

Software and code

Policy information about availability of computer code

Data collection	CytExpert v2.4; Nikon NIS-Element v5.02; Agilent Masshunter Qualitative and Quantitative Analysis vB.08.00, MiSeq Control Software v4.0
Data analysis	Data handling, statistics and data visualization were carried out in Microsoft Excel 2019 and R v4.1.1, using ggplot2 v.3.4.1, vegan v2.6.2, ggpubr v0.4.0 , dplyr v1.0.8, tidyr v1.1.3, tibble v.3.6. Raw amplicon sequences were trimmed with SeqtK v1.3, pair-end were merged with FLASH v1.2.3, adaptors were removed with CutAdapt v3.5, denoising, dereplicating and taxonomy assignment were done with DADA2 v1.22.0. Analysis of 16S rRNA sequences was performed on R v4.1.1 with phyloseq package and MicrobiomeAnalyst 2.0. Permutational multivariate analyses of variance (PERMANOVA) were carried out using Adonis function v2.0.4 on R v4.1.1. Assessment of enriched taxa in laminarin treatments compared to the surrounding seawater was done using ANCOM-BC package v2.02 on R v4.1.1.<br>Tracking analysis was done on Python v 3.11.0 with TrackPy v0.5.0. Mass spectrometry data was analyzed with Agilent Masshunter Qualitative and Quantitative Analysis vB.08.00.

For manuscripts utilizing custom algorithms or software that are central to the research but not yet described in published literature, software must be made available to editors and reviewers. We strongly encourage code deposition in a community repository (e.g. GitHub). See the Nature Portfolio guidelines for submitting code & software for further information.

Data

Policy information about availability of data

All manuscripts must include a data availability statement. This statement should provide the following information, where applicable:

- Accession codes, unique identifiers, or web links for publicly available datasets
- A description of any restrictions on data availability
- For clinical datasets or third party data, please ensure that the statement adheres to our policy

The amplicon sequencing data (16 rRNA gene) of the four isolates have been deposited on NCBI under accession numbers: OR501448-51 [https://www.ncbi.nlm.nih.gov/nuccore/OR501448.1/]; [https://www.ncbi.nlm.nih.gov/nuccore/OR501449.1/]; [https://www.ncbi.nlm.nih.gov/nuccore/OR501450.1/]; [https://www.ncbi.nlm.nih.gov/nuccore/OR501451.1/]. Raw amplicon fastq files were deposited in the Sequence Read Archive under accession number PRJNA1015554 [https://www.ncbi.nlm.nih.gov/bioproject/PRJNA1015554/]. Raw spectral files for in vitro enzyme assays have been deposited into the MassIVE database, with accession code MSV000092825 [doi:10.25345/C5VD6PF94]. All public sequences used for DMSP gene orthology analysis are available in the KEGG database. Source data are provided with this paper.

Human research participants

Policy information about studies involving human research participants and Sex and Gender in Research.

Reporting on sex and gender	Not human research
Population characteristics	Not human research
Recruitment	Not human research
Ethics oversight	Not human research

Note that full information on the approval of the study protocol must also be provided in the manuscript.

Field-specific reporting

Please select the one below that is the best fit for your research. If you are not sure, read the appropriate sections before making your selection.

☐ Life sciences ☐ Behavioural & social sciences ☒ Ecological, evolutionary & environmental sciences

For a reference copy of the document with all sections, see nature.com/documents/nr-reporting-summary-flat.pdf

Ecological, evolutionary & environmental sciences study design

All studies must disclose on these points even when the disclosure is negative.

Study description	This study aimed to characterize the ability of a natural marine bacterial community to chemotax towards the marine polysaccharides laminarin, alignates and their oligo- and monomeric building blocks, determine how environmental biochemical variables influence bacterial chemotactic responses and the underlying mechanisms in action. To do so, we carried out chemotaxis assays in situ and in laboratory conditions, flow cytometry, 16S rRNA amplicon sequencing of chemotactic communities, bacterial isolations from environmental samples, growth experiments, cell tracking and mass spectrometry measurements. Each experiment was replicated. The experiments were either one- or two-factors design.
Research sample	The research samples consisted of seawater from a coastal site containing natural communities of marine heterotrophic bacteria, representative of a natural population of marine bacteria in coastal environments. The study site is located within 100 m from a fully equipped laboratory, allowing for the direct analysis of the samples, repeated sampling and storage of samples. Escherichia coli K-12 was also used in laboratory conditions.
Sampling strategy	Replicates In Situ Chemotaxis Assays (ISCAs) were deployed simultaneously in coastal water near Bergen, Norway. ISCAs were deployed at 1 meter depth for two hours. Samples were then collected from ISCAs for downstream analyses. Pilot experiments in the field and in laboratory conditions were carried out before conducting the main experiment and are reported in the Supplementary information. We selected our sample size based on these pilot studies, the available literature (Lambert et al., 2017; Raina et al. 2022), feasibility and the variability between replicates. No statistical method was used to predetermine sample size.
Data collection	ISCA samples were collected in situ, and subsets were either snap frozen in liquid nitrogen or fixed in glutaraldehyde (done by E.E.C, B.S.L and J.-B.R.). Temperature, pH, salinity and oxygen concentrations were also recorded in situ with a CTD probe (done by A.V and F.V). Cell counts data generated from in situ sampling or laboratory experiments were recorded using CytExpert Version 2.4 (done by E.E.C and V.A.). Libraries for 16S rRNA gene sequencing were generated for all samples using the Nextera XT DNA Sample Preparation Kit (Illumina) and samples were sequenced with an Illumina Miseq platform (done by E.E.C). Cell tracking was performed on Nikon NIS-Element software with TrackPy v0.5.0 (done by J.M.K). Mass spectra were collected electronically using a Agilent 6546 Quadrupole Time of Flight Mass Spectrometer and the Agilent Masshunter Qualitative and Quantitative Analysis version B.08.00

software (done by S.P). DMSP concentration was measured on Shimadzu GC14A gas chromatograph (done by M.M.N and R.Si).

Timing and spatial scale	In situ chemotaxis experiments were performed once per day on days 0, 3, 9, 12, 15, 18 and 22 (note: day 0 marks the start of the measurements) during a phytoplankton bloom, with start date 28.05.2018 and end date 18.06.2018. These days were chosen to capture the different phases of the bloom, while being spaced by a relatively equal amount of time 3 to 3-4 days). For chemotaxis experiments in situ and in laboratory conditions, data were collected after one to two hours (based on previously published protocols). All chemotaxis experiments and sampling were performed at the same location to reduce spacial variability. For growth experiments on laminarin, oligomers and DMSP, data were collected every hour for 48h. For cell tracking, measurements were taken after 15 min, 40 min and 75 min after DMSP and/or laminarin amendment. For mass spectrometry measurements, data was collected every 2 min for 250 min.
Data exclusions	No data was excluded from the study and analyses.
Reproducibility	The in situ experiments described in the manuscript are part of a fully replicated environmental campaign carried out on different days. In situ experiments were carried out with at least three biological replicates. Laboratory-based experiments were conducted with three biological replicates, with all attempts at replication successful. All control tests were performed in triplicates.
Randomization	Treatment positions were fully randomized within the In Situ Chemotaxis Assay. Microorganisms were recruited in situ from seawater and therefore not preallocated into experimental groups.
Blinding	Blinding was not pertinent to our study because it did not include any animals and/or human research participants. In addition, blinding was not possible since many analyses were also carried out by the persons in charge of sampling.

Did the study involve field work? ☒ Yes ☐ No

Field work, collection and transport

Field conditions	Experiments were performed during 22 days in May-June 2018, in sunny conditions and at temperatures ranging between 15 and 20 °C. Rainfall only happened on day 20 of the campaign, and not data were recorded that day.
Location	The field experiments were performed at Espegrend Marine Research Field Station (60.2696097°S, 5.2233124°E), a coastal location near Bergen, Norway.
Access & import/export	Access to the field site and required permits were gained through the University of Bergen and the AQUACOSM project (VIMS-EHUX campaign).
Disturbance	The study created no disturbance to the ecosystem.

Reporting for specific materials, systems and methods

We require information from authors about some types of materials, experimental systems and methods used in many studies. Here, indicate whether each material, system or method listed is relevant to your study. If you are not sure if a list item applies to your research, read the appropriate section before selecting a response.

Materials & experimental systems

n/a	Involved in the study	
☒	☐	Antibodies
☒	☐	Eukaryotic cell lines
☒	☐	Palaeontology and archaeology
☒	☐	Animals and other organisms
☒	☐	Clinical data
☒	☐	Dual use research of concern

Methods

n/a	Involved in the study	
☒	☐	ChIP-seq
☐	☒	Flow cytometry
☒	☐	MRI-based neuroimaging

Flow Cytometry

Plots

Confirm that:

- [x] The axis labels state the marker and fluorochrome used (e.g. CD4-FITC).
- [x] The axis scales are clearly visible. Include numbers along axes only for bottom left plot of group (a 'group' is an analysis of identical markers).
- [x] All plots are contour plots with outliers or pseudocolor plots.
- [x] A numerical value for number of cells or percentage (with statistics) is provided.

Methodology

Sample preparation	Seawater samples derived from the In Situ Chemotaxis Assay field deployment were fixed (1% glutaraldehyde, final concentration) and snap frozen for shipment. Upon analysis, samples were snap thawn. Each sample was stained with SYBR Green (1:10,000 final dilution; ThermoFisher), incubated for 15 min in the dark and analyzed. Samples derived from laboratory-based were treated similarly, yet were not frozen but treated immediately after retrieval.
Instrument	CytoFLEX S (Beckman Coulter, USA)
Software	CytExpert Version 2.4
Cell population abundance	The target bacterial cell populations were the only ones present in the analysis plots.
Gating strategy	Gating around the bacterial populations was determined by pilot studies and analyzing bulk seawater.

- [x] Tick this box to confirm that a figure exemplifying the gating strategy is provided in the Supplementary Information.
